# Head-to-head Intra-individual Comparison of [^68^Ga]-FAPI and [^18^F]-FDG PET/CT in Patients with Bladder Cancer

**DOI:** 10.1007/s11307-022-01715-3

**Published:** 2022-03-29

**Authors:** E. Novruzov, K. Dendl, H. Ndlovu, P. L. Choyke, M. Dabir, M. Beu, F. Novruzov, E. Mehdi, F. Guliyev, S. A. Koerber, I. Lawal, G. Niegisch, J. Debus, U. Haberkorn, M. Sathekge, F. L. Giesel

**Affiliations:** 1 Department of Nuclear Medicine, Medical Faculty, Heinrich-Heine-University, University Hospital Dusseldorf, Dusseldorf, Germany; 2 Department of Nuclear Medicine, University Hospital Heidelberg, Heidelberg, Germany; 3 Department of Nuclear Medicine, University of Pretoria & Steve Biko Academic Hospital, Pretoria, South Africa; 4 Molecular Imaging Branch, National Cancer Institute, Bethesda, MD USA; 5 Nuclear Medicine Department, National Centre of Oncology, Baku, Azerbaijan; 6 Department of Uro-Oncology, National Centre of Oncology, Baku, Azerbaijan; 7 Department of Radiation Oncology, Heidelberg University Hospital, Heidelberg, Germany; 8 Department of Urology, University Hospital Duesseldorf, Duesseldorf, Germany

**Keywords:** FAPI, PET, Bladder cancer, Fibroblast activation protein, Urothelial carcinoma

## Abstract

**Aim/Purpose:**

Fibroblast activation protein-(FAP)-ligands, a novel class of tracers for PET/CT imaging, demonstrated promising results in previous studies in various malignancies compared to standard [^18^F]FDG PET/CT. ^68^Ga-labeled fibroblast activation protein inhibitor-([^68^Ga]Ga-DOTA-FAPI)-PET/CT impresses with sharp contrasts in terms of high tumor uptake and low background noise leading to clear delineation. [^18^F]FDG PET/CT has limited accuracy in bladder cancer due to high background signal. Therefore, we sought to evaluate the diagnostic potential of [^68^Ga]FAPI in patients with bladder cancer.

**Material and Methods:**

This retrospective analysis consisted of 8 patients (median age 66), 7 of whom underwent both [^68^Ga]FAPI and [^18^F]FDG PET/CT scans with a median time interval of 5 days (range 1–20 days). Quantification of tracer uptake was determined with SUV_max_ and SUV_mean_. Furthermore, the tumor-to-background ratio (TBR) was derived by dividing the SUV_max_ of tumor lesions by the SUV_max_ of adipose tissue, skeletal muscle, and blood pool.

**Results:**

Overall, 31 metastases were detected in five patients including lymph node metastases (n = 23), bone metastases (n = 4), lung metastases (n = 3), and a peritoneal metastasis (n = 1). In one patient, [^68^Ga]FAPI demonstrated significant uptake in the primary tumor located in the bladder wall. [^68^Ga]FAPI-PET/CT demonstrated significantly higher uptake compared to [^18^F]FDG PET/CT with higher mean SUV_max_ (8.2 vs. 4.6; p = 0.01). Furthermore, [^68^Ga]FAPI detected additional 30% (n = 9) lesions, missed by [^18^F]FDG. TBR demonstrated favorable uptake for [^68^Ga]FAPI in comparison to [^18^F]FDG. Significant differences were determined with regard to metastasis/blood pool ([^68^Ga]FAPI 5.3 vs [^18^F]FDG 1.9; p = 0.001).

**Conclusion:**

[^68^Ga]FAPI-PET/CT is a promising diagnostic radioligand for patients with bladder cancer. This first described analysis of FAP-ligand in bladder cancer revealed superiority over [^18^F]FDG in a small patient cohort. Thus, this so far assumed potential has to be confirmed and extended by larger and prospective studies.

**Supplementary Information:**

The online version contains supplementary material available at 10.1007/s11307-022-01715-3.

## Introduction

Urothelial carcinoma is the most common (> 90%) cell type of bladder cancer associated with worldwide highly varying incidence and prevalence rates mainly depending on environmental factors [1–4]. While bladder cancer in its early clinical stages (non-muscle-invasive bladder cancer, NMIBC ≤ T1) has an overall good prognosis, it nevertheless is associated with high recurrence rates (40–70% rate within 5 years). The prognosis in advanced clinical stage disease (muscle-invasive bladder cancer; MIBC ≥ T2) is poor due to the early development of distant metastases. Accurate staging plays a crucial role for proper patient stratification and therapy management [5, 6].

Besides T-stage, nodal status is the most important prognostic factor that correlates with 5-year disease-free survival. Furthermore, the extent of nodal metastasis shows a direct correlation with T-status at the time of initial presentation which reveals a lymph node involvement of approximately 30% in T2 and up to 60% in ≥ T3 cancers [7]. The imaging modalities of computed tomography urography (CTU) and multiparametric magnetic resonance imaging (mpMRI) along with the urological examination methods provide a clinically acceptable diagnostic performance in patients with early clinical stages (NMIBC), whereas the diagnostic performance of the conventional imaging modalities for initial tumor staging has been disappointing due to their low sensitivity for lymph node involvement (≤ 50%) in patients with advanced clinical stages (MIBC) (7–9). Although 2-deoxy-2-[^18^F]fluoro-D-glucose positron emission tomography/computed tomography ([^18^F]FDG PET/CT) is substantially better than conventional imaging modalities in tumor surveillance and therapy response monitoring, its diagnostic performance in detection of lymph node involvement for initial tumor staging in advanced clinical stages is only slightly better than conventional imaging (up to 56%) [7, 8]. The renal clearance and high tracer accumulation in the urinary bladder are further limiting factors for the use of [^18^F]FDG PET/CT in primary tumor detection despite techniques such as urinary catheterization and administration of diuretics to reduce bladder activity [9–11].

Cancer-associated fibroblasts (CAFs) in the tumor microenvironment enhance pro-tumorigenic effects in many cancers including bladder cancer, which influences as a part of supportive tumor stroma various aspects of tumor development and progression as well as therapeutic response. CAFs promote tumorigenesis in urothelial bladder carcinoma via multiple markers including alpha smooth muscle actin (ASMA), CD90/Thy-1, fibroblast activation protein (FAP), platelet derived growth factor receptor-alpha and -beta (PDGFR-α/-β) and especially in advanced stages significantly increased FAP-expression [12, 13]. FAP is a type II transmembrane serine protease with post proline dipeptidyl peptidase as well as endopeptidase activity and its increased expression appears to be an independent adverse prognostic factor in urothelial bladder cancer [13]. Similar correlations are observed in breast cancer, colorectal cancer, ovarian cancer, and pancreatic ductal adenocarcinoma. FAP-expression is also observed in chronic inflammation and fibrotic diseases [14, 15].

Thus, [^68^Ga]FAPI, a novel, recently developed tracer family targeting FAP shows a favorable tumor-to-background ratio and some important practical advantages regarding the scan preparations. Therefore, [^68^Ga]FAPI in some cancer types may be even superior to [^18^F]FDG regarding diagnostic sensitivity, specificity, and potentially theranostic application [16–20].

This multicenter, retrospective pilot analysis aimed to evaluate the potential role of [^68^ Ga]FAPI-PET/CT in the assessment of bladder cancer in comparison with [^18^F]FDG in patients with bladder cancer regarding biodistribution and tumor uptake.

## Materials and Methods

### Patient cohort

Eight male patients were recruited from three centers (University Hospital Pretoria *n* = *4*, Azerbaijan National Centre of Oncology *n* = *3,* and University Hospital Heidelberg *n* = *1*) with histopathologically confirmed therapy-naïve or pre-treated bladder cancer (early and advanced stages). These patients underwent both [^18^F]FDG- and [^68^Ga]FAPI-PET/CT scans with a median of 5 days (range 1–20) apart between March and June 2021. The data were then anonymized, centralized, and retrospectively analyzed at University Hospital Duesseldorf (UKD). The [^18^F]FDG PET/CT scans were conducted based on oncological standard care of indications. [^68^Ga]FAPI-PET/CT was performed on the same patients on an individual patient basis after obtaining written informed consent following national regulations, Good Clinical Practice (GCP) and the Declaration of Helsinki.

### PET image acquisition

Imaging data was acquired 60 min after tracer application and whole body images encompassing from the head to mid-thigh for both [^18^F]FDG PET/CT and [^68^Ga]FAPI PET/CT. All PET scans were performed in 3D mode with an acquisition time of 3–5 min/bed position at all sites. All patients were monitored for adverse events up to 30 min after the end of the examination. The median time interval between [^18^F]FDG PET/CT and [^68^Ga]FAPI PET/CT patients (n = 7) was 5 days (range 1–20 days). There was no change in therapy between the scans.

#### ***[***^***18***^***F]FDG PET Imaging***

Following a fasting time of 6 h, [^18^F]FDG is administered (median activity 300 MBq, range of 207–385) at serum glucose levels of < 130 mg/dl.

#### ***[***^***68***^*** Ga]FAPI PET Imaging***

Two different ^68^Ga-labeled FAP ligands with similar chemical composition were used in this study (median injected activity 148 MBq, range 63–185), [^68^Ga]FAPI-04: n = 5, [^68^Ga]FAPI-46: n = 3. Radiosynthesis and labeling were performed as described previously for all ^68^Ga-labeled FAP ligands [21, 22].

### Image analysis

Tracer uptake was quantified by mean and maximum standardized uptake values (SUV_mean_ and SUV_max_) for both [^68^Ga]FAPI and [^18^F]FDG PET/CT scans. SUV values were determined by drawing volumes of interest (VOIs) on metastatic lesions observed with [^18^F]FDG and [^68^Ga]FAPI. Volumes of interest (VOI) were placed over the normal organs by one UKD investigator (EN; supervised by FLG) with a diameter of 1 cm for small organs (thyroid, parotid gland, myocardium, oral mucosa, and spinal cord) or 2 cm for other organs (brain, muscle, liver, spleen, kidney, fat, aortic lumen, and lung). Circular regions of interest (ROI) were placed on axial slices around lesions with focally increased tracer uptake and were automatically incorporated into a 3-dimensional volume of interest (ESoft; Siemens). A 40% iso- contouring approach was used for organs as well as lesions. Only unequivocally positive lesions on the [^18^F]FDG or [^68^Ga]FAPI scan, whether primary or metastatic, were considered to be suitable for further analysis. The lesions are classified as unequivocally positive, when the SUV_max_ is more than 3 times that of the blood pool. Tumor-to-background ratios (TBRs) were determined to quantify the image contrast. TBR was calculated by dividing the SUV_max_ of the metastases by background values (blood pool, fat tissue and skeletal muscle).

### Statistical analysis

We used descriptive analyses for demographics, tumor characteristics and tracer uptake. Comparison between [^68^Ga]FAPI- and [^18^F]FDG PET/CT-SUV metrics in tumor and normal tissue as well as TBRs was performed with the paired t-test and Wilcoxon-Mann–Whitney-Test. A p value of < 0.05 was considered statistically significant. All statistical analyses were performed using SigmaStat Version 3.5 (Systat Software, Inc., San Jose, CA, USA) and SigmaPlot Version 11.0 (Systat Software, Inc., San Jose, CA, USA) for graphical visualization.

## Results

### Study population

Eight male patients were included in the study with a median age of 66 years (range 57–78). Four of them had been newly diagnosed with bladder cancer, while four patients were under evaluation for recurrent disease with advanced tumor stage. Two of the patients with newly diagnosed bladder cancer were advanced stage (IIIA and IVB), while the other two patients had localized disease (Stage I). Both of the latter two patients demonstrated a local, intracystic lesion of NMIBC, which is consistent with the previous studies that patients with NMIBC are much less likely to exhibit metastatic lesions (5, 6). The patients with recurrent bladder cancer had received either a local BCG-Therapy (intravesical Bacillus Calmette-Guérin immunotherapy) following a TUR-B-(Transurethral resection of bladder tumor)-Resection or a radical cystoprostatectomy with a neobladder reconstruction. In total, we detected 31 metastatic lesions in five patients (median metastatic lesion 7, range 2–11).

### Biodistribution in normal organs

**Fig. 1:**
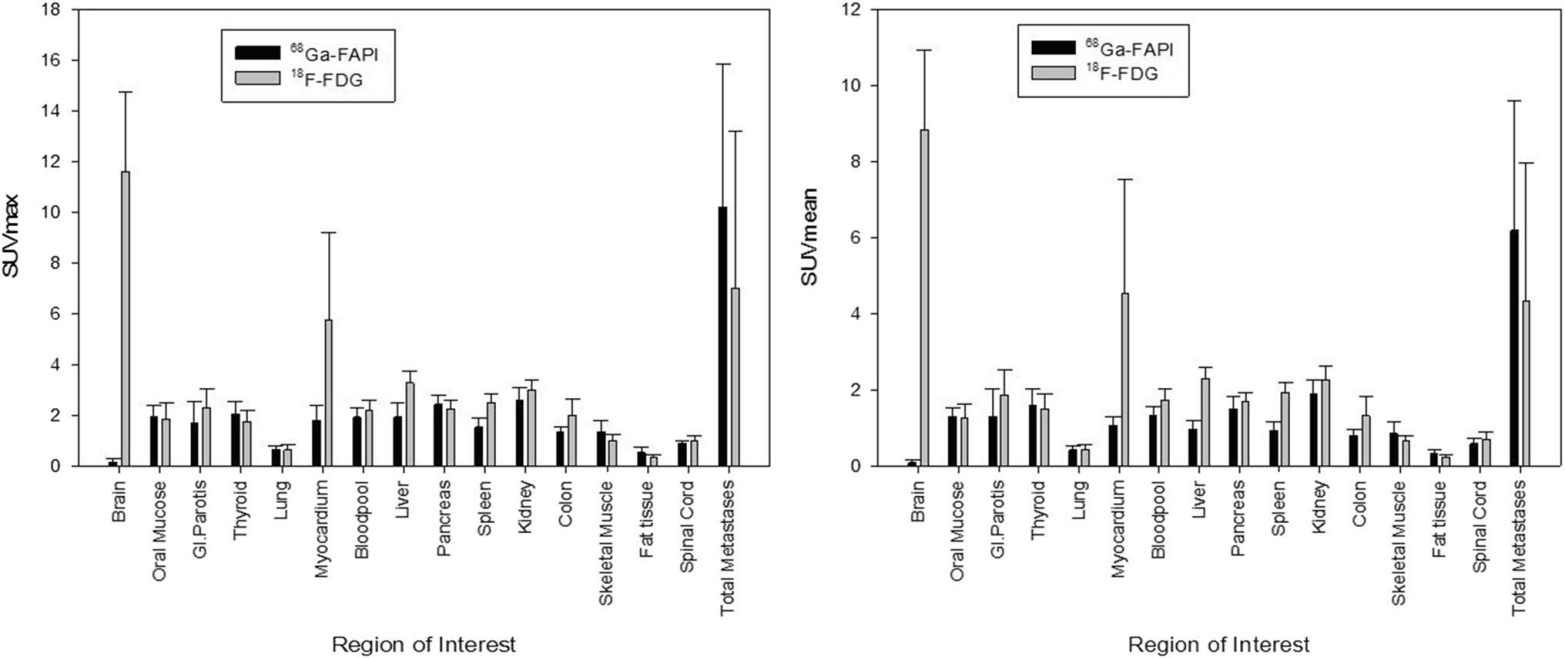
Biodistribution (SUV_max_ and SUV_mean_) of [^68^ Ga]FAPI in comparison to [^18^F]FDG in normal organs and metastatic lesions (mean values and standard deviations)

The biodistribution of [^68^Ga]FAPI and [^18^F]FDG is presented in Fig. 1. The background activity of [^68^Ga]FAPI was lower than [^18^F]FDG in most normal tissues (11 of 15 organs). Our patient cohort showed a statistically significant lower [^68^ Ga]FAPI-uptake in brain parenchyma, myocardium, liver, spleen, blood pool and kidney cortex (p < 0,05). These results appear to be in line with previous studies [18–36].

### Uptake in tumor lesions and tumor-to-background ratio (TBR)

**Fig. 2:**
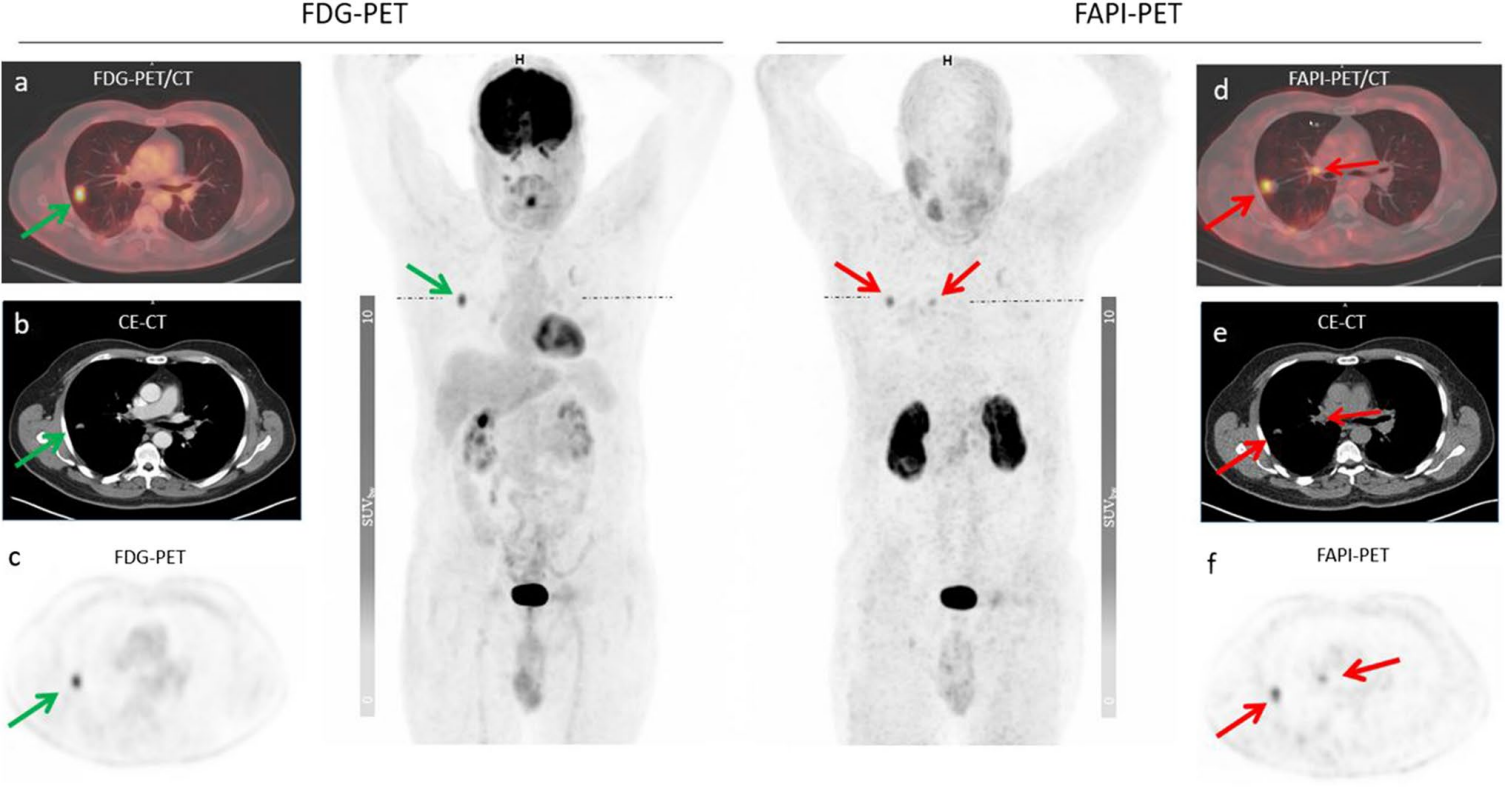
A 60-year-old patient in the 7^th^ year of follow-up for bladder cancer underwent restaging due to a suspicious lung finding on the right side. The PET/CT scan shows [^68^ Ga]FAPI avid hilar lymph node metastasis (red arrow) as well as both [^68^ Ga]FAPI and [^18^F]FDG avid pulmonary metastasis in the right lung. The quantified uptake in the hilar lymph nodes on [^68^ Ga]FAPI (d -f) was SUV_max_ 5,5 compared to the [^18^F]FDG uptake (a–c.) with an SUV_max_ 1,4 and in the lung metastasis (green arrow) on the right side on [^68^ Ga]FAPI was SUV_max_ 7,0 compared to the [^18^F]FDG uptake with an SUV_max_ 6,3 (green arrow = [^18^F]FDG, red arrow = [^68^Ga]FAPI)

**Fig. 3:**
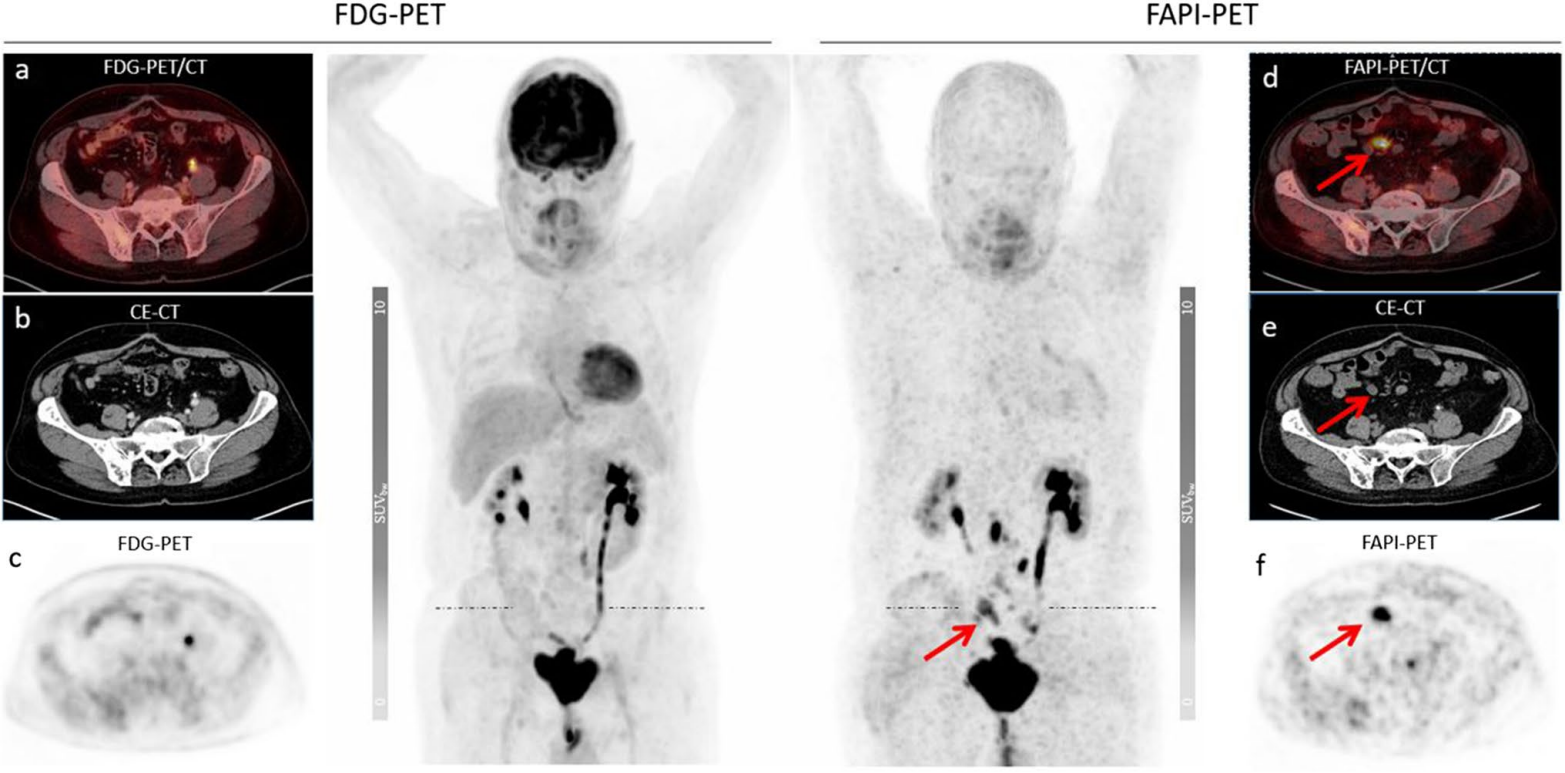
68-year-old patient in the 2^nd^ year of the follow-up after radical cystectomy with local recurrence und restaging with PET/CT scan. The PET/CT scan reveals multiple enlarged abdominal lymph node metastases, i.e., mesenteric lymph node metastases, which are only [^68^ Ga]FAPI-avid (d–f) [^68^ Ga]FAPI-SUV_max_ 10,11 vs. [^18^F]FDG-SUV_max_ 3,16) (red arrow = FAPI)

**Fig. 4:**
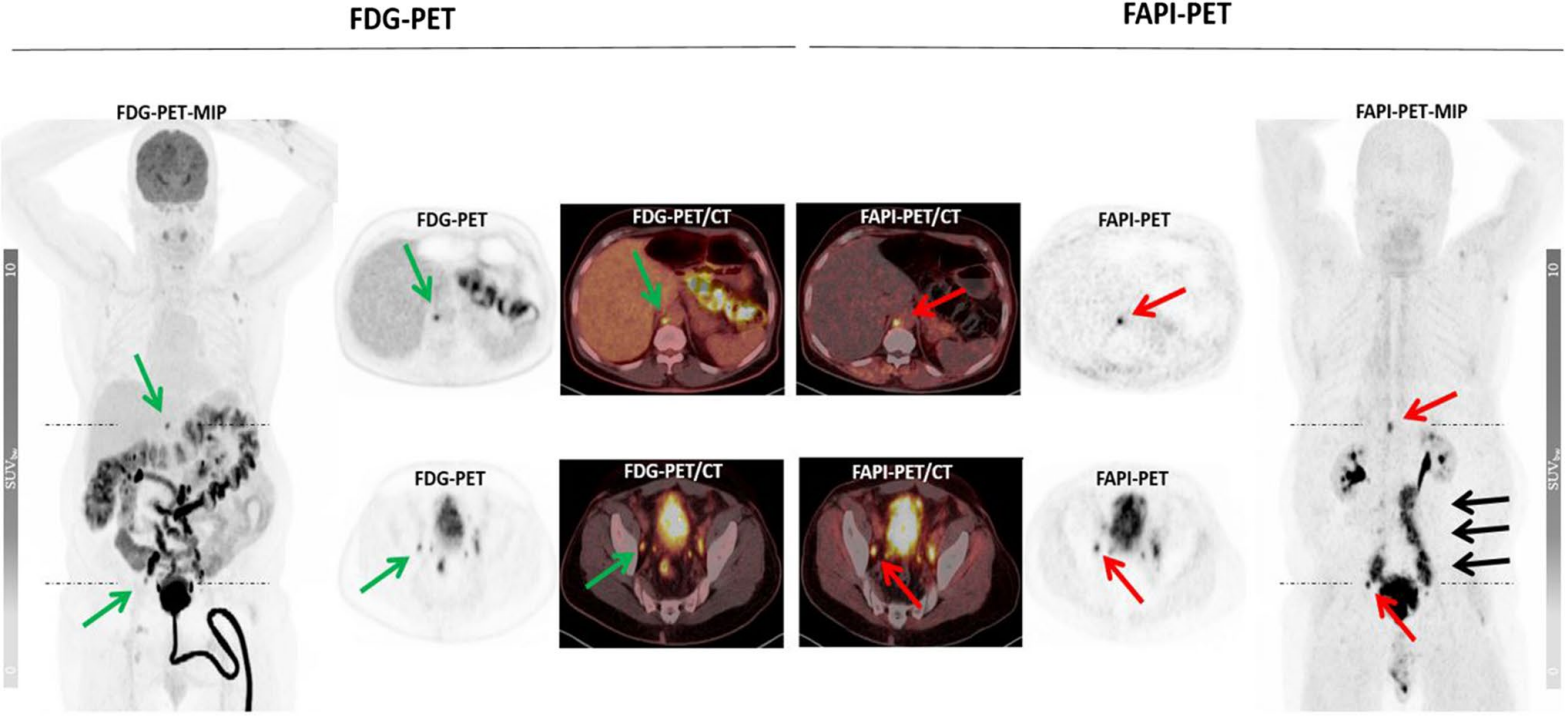
Intraindividual comparison of [^18^F]FDG and [^68^Ga]FAPI in a 65-year-old patient presenting with a strong [^68^ Ga]FAPI uptake in retroperitoneal/paraaortal ([^68^Ga]FAPI-SUV_max_ 12,0 vs. [^18^F]FDG-SUV_max_ 10,42) and pelvic metastatic lymph nodes ([^68^Ga]FAPI-SUV_max_ 15,28 vs. [^18^F]FDG-SUV_max_ 8,89) while only mild to moderate uptake on [^18^F]FDG PET/CT. The low [^68^Ga]FAPI uptake in normal organs and excellent tumor-to-background ratio lead to significantly better delineation of tumor lesions (black arrow) ([^68^Ga]FAPI-SUV_max_ 8,16 vs. [^18^F]FDG-SUV_max_ 3,86) (green arrow = [^18^F]FDG, red arrow = [^68^Ga]FAPI)

**Fig. 5:**
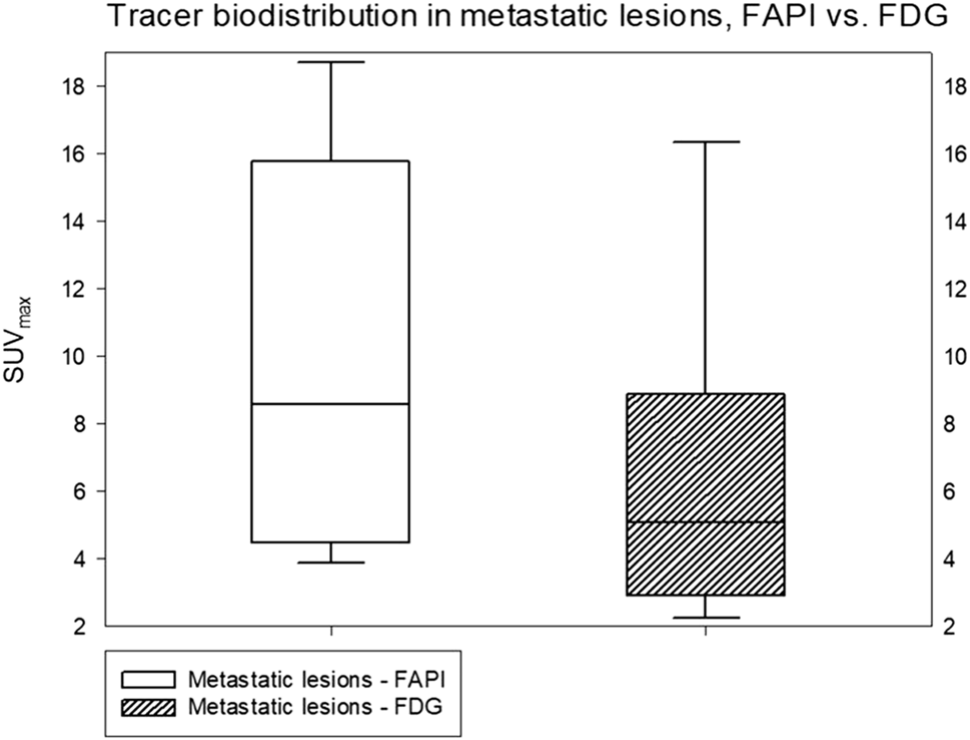
The depiction of overall metastatic lesions in a box-plot, FAPI vs. FDG

Since in patients with intraindividual comparison no unequivocally primary tumor could be detected, all lesions evaluated in this group were metastatic lesions. Impressively, [^68^Ga]FAPI demonstrated significantly higher accumulation compared to [^18^F]FDG (median SUV_max_ 8.16 vs. 4.64, p = 0.01). Furthermore, nine additional lesions were detected with [^68^ Ga]FAPI PET/CT, representing a 30% increased detection rate (n = 22 vs. n = 31) (Table 1). The comparison of uptake of metastatic lesions is depicted in Fig. 5. In one example, a 78-year-old patient undergoing [^68^Ga]FAPI PET/CT for staging, demonstrated high [^68^ Ga]FAPI uptake in the primary tumor located in the bladder wall. However, this case lacked a comparative [^18^F]FDG scan. Examples of [^68^Ga]FAPI-avid metastatic lesions that were [^18^F]FDG negative are depicted in Figs. 2 and 3. In addition, most patients showed marked differences between FAP and [^18^F]FDG in terms of tumor uptake and background activity, in both cases favoring FAP-ligands. An example of a patient with metastatic lesions demonstrating low [^18^F]FDG but strong [^68^Ga]FAPI uptake is illustrated in Fig. 4.

**Table 1:** The number of detected metastatic lesions by the tracers, [^18^F]FDG vs. [^68^ Ga]FAPI

	**[**^**18**^**F]FDG**	**[**^**68**^** Ga]FAPI**
*Lymph node*	15 (SUV_max_: 7,25 ± 6,67)	23 (SUV_max_: 10,58 ± 5,80)
*Bone*	4 (SUV_max_: 8,79 ± 5,28)	4 (SUV_max_: 5,56 ± 2,97)
*Lung*	3 (SUV_max_: 9,56 ± 5,61)	3 (SUV_max_: 8,63 ± 5,58)
*Peritoneum*	0 (SUV_max_: 1,46)	1 (SUV_max_: 8,13)
*Total metastatic lesions*	22	31

All TBRs demonstrated higher values and, thereby, sharper contrasts, in terms of low background activity and strong uptake of malignant tissue for FAPI in comparison to [^18^F]FDG (Fig. 5). However, only regarding tumor/blood pool significant differences were determined ([^68^Ga]FAPI vs. [^18^F]FDG, 5.33 vs. 1.95, p = 0.001) (Table 2).

**Table 2:** Tumor-to-background ratio (TBR) of metastatic lesions (p < 0.05)

VOI	Tumor/blood pool		Tumor/skeletal muscle		Tumor/fat tissue
Tracer	[^68^ Ga]-FAPI	[^18^F]-FDG	[^68^ Ga]-FAPI	[^18^F]-FDG	[^68^ Ga]-FAPI [^18^F]-FDG
TBR	5.33	1.95	3.63	2.80	16.93 14.86
P value	**0.001**		0.278		0.632

## Discussion

This pilot study shows in a small cohort of patients that [^68^Ga]FAPI may be more sensitive for bladder cancer metastases than [^18^F]FDG. TNM staging of bladder cancer is challenging with conventional imaging modalities and new methods are needed. One potential reason for the significantly higher uptake with [^68^Ga]FAPI-uptake compared to [^18^F]FDG, might be due to the abundant tumor stroma in bladder cancer with FAP-overexpression of CAFs depending on the tumor stage [12, 13]. Prior studies of [^68^Ga]FAPI have shown advantages to this agent in breast cancer, colorectal cancer, ovarian cancer and pancreatic ductal adenocarcinoma. Therefore, these results are in line with previously published results [16–18, 23–36].

Staging of bladder cancer currently employs several conventional imaging tools. CT Urography (CTU) is the method of choice for primary bladder cancer staging but has relatively poor soft tissue resolution for muscular invasion and also a very low sensitivity for lymph node involvement. In light of the limitations of CTU, mpMRI is considered to be an alternative method for local staging of bladder cancer because of its higher soft-tissue contrast resolution. Although it can assess muscular invasion more successfully, it also has very limited sensitivity and specificity for nodal involvement. Bladder cancer can be detected with [^18^F]FDG PET/CT scans; however, it has been of limited use for primary staging of bladder cancer due to high renal clearance and tracer accumulation in the urinary bladder. However, [^18^F]FDG PET/CT imaging is relatively insensitive for nodal involvement [7]. Since lymph node involvement directly correlates with T-stage, differentiation of T2 and ≥ T3 tumor is essential for accurate interpretation of [^18^F]FDG PET/CT. Thus, conventional imaging modalities such as mpMRI, CTU, and [^18^F]FDG PET/CT are relatively poor at establishing nodal status in primary tumor staging which has implications for therapy planning and prognosis in patients with bladder cancer [7, 8].

In comparison to [^18^F]FDG, [^68^ Ga]FAPI demonstrates lower background noise and higher target signal (measured in SUV_max_ and SUV_mean_), thus improving detection rate. Up to 30% more metastatic lesions were detected compared to [^18^F]FDG. Due to high local recurrence rates in NMIBC and the importance of T-staging in advanced bladder cancer (MIBC), it would be of interest to determine if [^68^Ga]FAPI could improve T-staging in primary tumor diagnosis and for tumor surveillance of early bladder cancer recurrence [3–5]. In the primary setting, improved tumor delineation by [^68^Ga]FAPI would facilitate planning of treatment in cases with high grade carcinoma in situ or locally advanced disease. However, [^68^ Ga]FAPI, like [^18^F]FDG, is excreted via urinary tract leading to high background within the bladder, thereby masking uptake in the primary tumor and requiring additional measures such as forced diuresis and/or voiding before scanning. Therefore, it is expected that the one patient in our series in whom it was possible to identify the primary tumor, is an unusual event, however, this needs to be confirmed in a larger cohort. However, with regard to N staging, this study suggests more promising results and may find a role in initial staging or recurrence.

Although different equipment and imaging protocols were utilized at the three centers involved, the findings were consistent across sites (Supplementary Table 1), particularly in terms of detected metastatic lesions based on increased tumor uptake as well as a significantly higher tumor-to-background ratio (SUV_max_ tumor/blood pool). Since the most frequently used FAPI-ligands [^68^Ga]FAPI-04 and [^68^Ga]FAPI-46 are considered to be interchangeable due to comparable biodistribution at 1 h p.i. and similar diagnostic performance so that the use of both agents in this study is not thought to be a problem [19, 23].

[^68^Ga]FAPI-based tumor assessment may improve treatment monitoring, especially after immune checkpoint inhibitors. It is well known that [^18^F]FDG may result in false positives due to pseudoprogression. As [^68^Ga]FAPI PET reflects the population of CAFs in the tumor microenvironment and CAFs are thought to decrease in number during response to therapy, it may be a better method of assessing patients thought to have pseudoprogression.

There are several limitations to this study, particularly its small size. Additionally, no histopathological verification was possible for lesions other than primary bladder lesions. In light of the important role of the histopathological subtypes of bladder cancer for the prognosis, we cannot perform a correlation between tumor grading and [^68^Ga]FAPI uptake due to the small patient cohort. Furthermore, an analysis of TBR and specific tracer uptake of the various metastatic lesions could not be performed.

## Conclusion

To our knowledge, this is the first investigation to evaluate the potential of FAP-ligands in bladder cancer. [^68^Ga]FAPI PET/CT is superior to [^18^F]FDG PET/CT in detecting metastatic lesions in patients with advanced bladder cancer. [^68^Ga]FAPI impresses with significantly higher uptake in tumor lesions and lower background activity, and therefore a highly promising theranostic agent for urological cancer diseases. Further, prospective research studies with larger patient cohorts are needed in order to elaborate sensitivity and specificity of FAP-ligands in bladder cancer.

## Supplementary information

Below is the link to the electronic supplementary material.

Supplementary file1 (35.1 KB)

## Data Availability

The data used and/or analyzed during the current study are available from the corresponding author on reasonable request.
