# Supplementary material for: Head-to-head Intra-individual Comparison of [68Ga]-FAPI and [18F]-FDG PET/CT in Patients with Bladder Cancer
Source: Mol Imaging Biol. 2022 Mar 29;24(4):651–8. doi: 10.1007/s11307-022-01715-3 (PMC9296390; doi:10.1007/s11307-022-01715-3)
Supplement: Supplementary file 1 — Supplementary file1 (35.1 KB) [file 11307_2022_1715_MOESM1_ESM.docx]

**Supplementary information**

Supplementary Table 1

| Site | University Hospital Pretoria | Azerbaijan National Center of Oncology | University Hospital Heidelberg |
| --- | --- | --- | --- |
| PET/CT scanner | Biograph mCT 40 slice, Siemens | Biograph mCT 40 slice, Siemens | Biograph mCT Flow, Siemens |
| Injected activity (MBq), ^68^Ga-FAPI | 148 – 185 | 63 - 152 | 200 |
| Injected activity (MBq), ^18^F-FDG | 207 - 385 | 296 - 322 | N/A |
| Time interval (median days) | 3 | 10 | N/A |
| CT reference (mAs) | 30 | 40 – 250 | 30 |
| CT peak kilovoltage (kV) | 130 | 120 | 130 |
| CT slice thickness (mm) | 5 | 3 | 5 |
| CT slice increment (mm) | 5 | 3 | 3 – 4 |
| PET reconstruction | OSEM algorithm | PSF + TOF | OSEM algorithm |
| Iterations | 2 | 2 | 2 |
| Subsets | 21 | 21 | 21 |
| Matrix | 200 x 200 | 200 x 200 | 200 x 200 |
| Corrections | Gaussian filter applied at FWHM of 5.0 mm | Gaussian FWHM 5.0 mm | Gaussian FWHM 5.0 mm |
